# Trends in thyroid surgery in Japan from 2014 to 2023: report on the National Clinical Database

**DOI:** 10.1007/s00595-025-03126-7

**Published:** 2025-11-17

**Authors:** Yoko Omi, Hiroyuki Yamamoto, Naoyoshi Onoda, Chisato Tomoda, Takahiro Okamoto, Shinichi Suzuki, Hisato Hara, Iwao Sugitani

**Affiliations:** 1 Department of Endocrine Surgery, Tokyo Women’s Medical University, 8-1 Kawada-Cho, Shinjuku-Ku, Tokyo, 162-8666 Japan; 2 Department of Healthcare Quality Assessment, Graduate School of Medicine, The University of Tokyo, 7-3-1 Hongo, Bunkyo-Ku, Tokyo, 113-8654 Japan; 3 Department of Surgery, Kuma Hospital, 8-2-35 Shimoyamatedori, Chuo-Ku, Kobe-Shi, Hyogo, 650-0011 Japan; 4 Department of Surgery, Ito Hospital, 4-3-6 Jingu-Mae, Shibuya-Ku, Tokyo, 150-8308 Japan; 5 Mirraza Shinjuku Tsurukame Clinic, Mirraza Shinjuku 7 F 3-36-10 Shinjuku, Shinjuku-Ku, Tokyo, 160-0022 Japan; 6 Department of Surgery, Japan Community Healthcare Organization (JCHO) Nihonmatsu Hospital, 1 Kanayagawa, Fukushima, 960-1296 Japan; 7 Department of Thyroid and Endocrinology, School of Medicine, Fukushima Medical University Hospital, 1589-3 Kashiwada-Cho Ushiku-Shi, Ibaraki, 300-1211 Japan; 8 Breast and Endocrine Surgery, Tsukuba Central Hospital, 1-1-5 Sendagi Bunkyo-Ku, Tokyo, 113-8602 Japan; 9 Department of Endocrine Surgery, Nippon Medical School, Bunkyō, Japan; 10 The Task Force on Annual Reporting of Thyroid Surgery of the Japan Association of Endocrine Surgery, using the National Clinical Database, Tokyo, Japan

**Keywords:** National Clinical Database, Thyroid surgery, Papillary thyroid cancer

## Abstract

**Purpose:**

Endocrine surgery encompasses various organ systems, but thyroid surgery represents the major component. The Japanese Association of Thyroid Surgeons published nationwide data on thyroid cancer surgery until 2005, since when no further reports have been published. Thus, we analyzed thyroid surgery trends using data from the National Clinical Database (NCD) from 2014 to 2023.

**Methods:**

We analyzed cases of thyroid surgery, for malignant and benign conditions, recorded in the NCD. A detailed analysis of papillary thyroid carcinoma (PTC) cases was conducted in 2014, 2019, and 2023 to assess TNM classification, surgical procedures, complications, and outcomes.

**Results:**

The number of thyroid surgery facilities and the number of thyroid surgeries being performed have both declined since 2020. A trend toward more hemithyroidectomies and fewer total thyroidectomies for PTC is evident. The number of surgeries for cancers < 1 cm and lateral node dissection for PTC have also decreased. Endoscopically assisted surgery has increased continuously, accounting for > 4% of thyroid operations in 2020. The incidence of permanent hypoparathyroidism has decreased and perioperative mortality is rare.

**Conclusion:**

The changes in surgical trends are aligned with guideline updates and shifts in surgical practice. Future efforts will refine data collection and utilize the NCD to improve thyroid surgical quality and research.

**Supplementary Information:**

The online version contains supplementary material available at 10.1007/s00595-025-03126-7.

## Introduction

Endocrine surgery targets organs throughout the body, including the thyroid gland, parathyroid gland, adrenal gland, breast, prostate, and endocrine tumors of the digestive tract. The surgical treatment of thyroid diseases is a major component of endocrine surgery and is performed not only for malignant tumors but also for benign diseases such as Graves’ disease and follicular nodular disease. The Japanese Association of Thyroid Surgeons (JATS) collected nationwide data on thyroid cancer surgery and included it in the annual abstract booklets of the society, although it was not formally published in academic journals. However, this was discontinued during the reorganization of JATS into the Japanese Society of Thyroid Surgery in 2005, and in 2018, the society merged with the Japanese Association of Endocrine Surgery (JAES). Since then, no reports have been published on the actual state of thyroid surgery in Japan.

The National Clinical Database (NCD) was established in 2010, and registration began in January, 2011. The NCD database project [1] covers > 95% of surgeries performed by general surgeons in Japan. Over 1.5 million surgical procedures are performed annually, and more than 1.57 million surgical procedures were collected from 5754 facilities in 2023 [2].

Data registered in the NCD are being utilized to improve the quality of surgical care in the fields of gastrointestinal surgery [3], cardiovascular surgery, thoracic surgery [4], pediatric surgery [5], and breast surgery [6]. As part of the feedback on NCD data entry to endocrine surgeons, we report an overview of thyroid surgery data entered from 2014 to 2023.

## Materials and methods

The study population comprised patients who underwent one or more thyroid surgical procedures, and whose surgical data were recorded in the NCD system between 2014 and 2023. Data were extracted using a secure system with no external connections, and basic statistical analyses were performed by NCD statisticians. To analyze the surgical status for papillary thyroid carcinoma (PTC), we extracted cases for which only “papillary carcinoma” was selected as the diagnosis at the time of admission, and analyzed the preoperative TNM classification, surgical procedure, postoperative pathological diagnosis, complications, and outcome at the time of discharge. The surgical treatment for PTC was analyzed in detail only for the years 2014, 2019, and 2023. These 3 years were selected because they represent the situation before and after revision of the guidelines in 2018 [7] and after the COVID-19 pandemic.

Cases in which several operative methods were performed simultaneously were tallied according to the individual operative methods. If multiple histological types were selected for the diagnosis at the time of admission, all entries for each type were tabulated. Surgical mortality included 30 day and in-hospital mortality, and the NCD system ensured follow-up during hospitalization for at least 90 days after surgery.

This study was approved by the Ethics and Conflict of Interest Committee of the NCD [https://www.ncd.or.jp/about/pdf/ethics_review_application_20231010.pdf (in Japanese); https://www.ncd.or.jp/about/pdf/ethics_review_report_20231010.pdf (in Japanese)].

## Results

### Overview of thyroid surgery

**Fig. 1:**
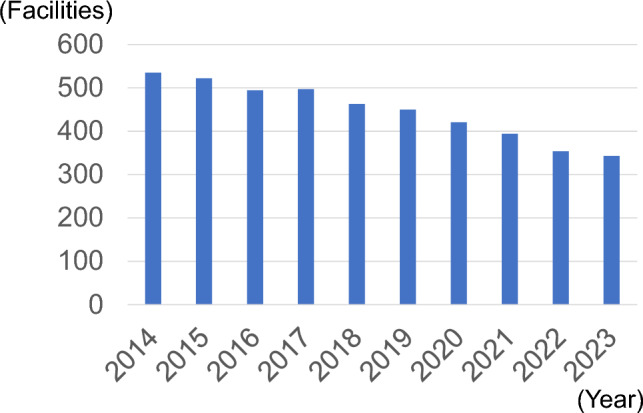
Number of participating facilities that registered thyroid surgeries from 2014 to 2023

**Fig. 2:**
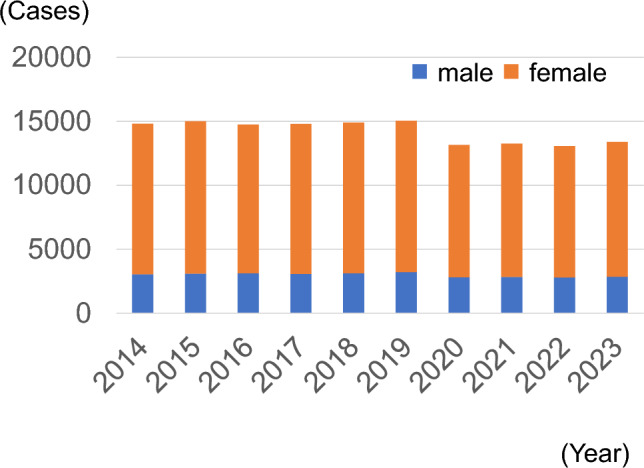
Total number of thyroid surgeries registered in the National Clinical Database from 2014 to 2023, according to gender

**Fig. 3:**
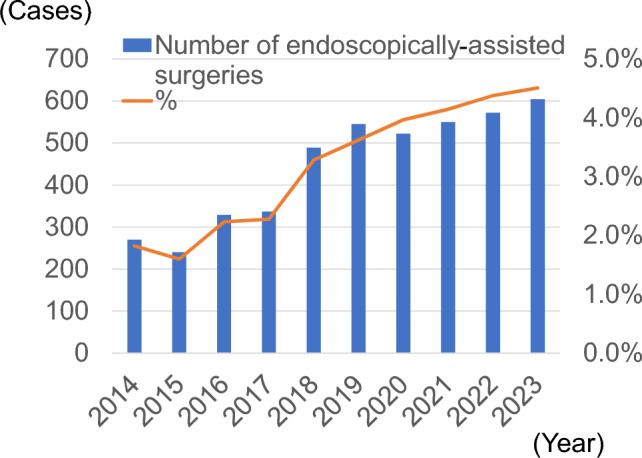
Number of endoscopically assisted surgeries registered in the National Clinical Database from 2014 to 2023. The number of surgeries is shown on the left axis and the proportion of the total number of surgeries is shown on the right axis

**Fig. 4:**
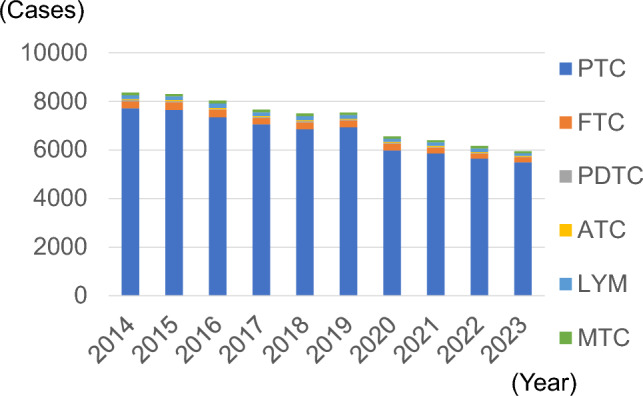
Number of surgeries for malignant tumors according to the histological subtypes. *PTC* papillary thyroid carcinoma, *FTC* follicular thyroid carcinoma, *PDTC* poorly differentiated thyroid carcinoma, *ATC* anaplastic thyroid carcinoma, *LYM* lymphoma, *MTC* medullary thyroid carcinoma

**Fig. 5:**
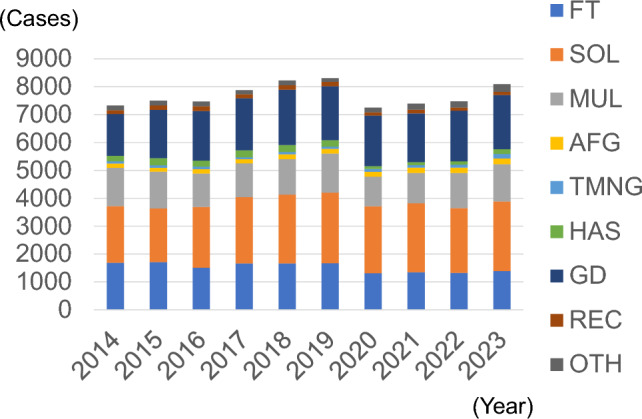
Number of surgeries for benign diseases according to the diagnosis on admission. *FT* follicular tumor, *SOL* solitary nodule, *MUL* multiple nodule, *AFG* autonomous functioning goiter, *TMNG* functioning multinodular goiter, *HAS* Hashimoto’s disease, *GD* Graves’ disease, *REC* recurrent disease, *OTH* others

Figure 1 shows the trend in the number of facilities that registered thyroid surgeries. The number of registered facilities declined over time. By 2023, there were 343 facilities. Figure 2 shows the trend in the number of registered thyroid surgeries. There was no significant change from 2014 to 2019, but it decreased by approximately 10% after 2020. The male to female ratio was approximately 1:4, with no change observed. Figure 3 shows the trends in endoscopically assisted thyroidectomy. The number of endoscopically assisted thyroidectomies has increased over the years, accounting for > 4% of all surgeries since 2020. Table 1 presents an overview of the number of thyroid surgery registries. Figure 4 shows the trend in surgical cases registered for a diagnosis of a malignant tumor at the time of admission. Although there was a declining trend over the years, there was no significant change in the histological types of disease at the time of patient admission. The percentages for the entire year were as follows: papillary thyroid carcinoma, 91.8%; follicular thyroid carcinoma, 3.7%; poorly differentiated thyroid carcinoma, 0.5%; anaplastic thyroid carcinoma, 0.6%; lymphoma, 2.1%; and medullary thyroid carcinoma, 1.3%. It is important to note that these were the names of the diseases at the time of admission and differed from the final histopathological diagnosis. Figure 5 shows the trend for surgical cases of lesions not diagnosed as malignancy at the time of admission. There was a trend toward an increase until 2019, but in 2020, there was a decrease of approximately 13%. After that, there was a trend toward an increase again, and in 2023, the number of cases was almost the same as that in 2019. There were no significant changes in the name of the disease at the time of admission.

**Table 1:** Overview of thyroid surgeries registered in the National Clinical Database

Year	Participating facilities	Surgeries	Male	Median age (25–75th percentile)	Female	Median age (25–75th percentile)	Endoscopically assisted surgeries
2014	535	14,812	3045	56 (43–67)	11,767	54 (41–66)	270
2015	522	15,014	3104	55 (41–66)	11,910	54 (41–66)	240
2016	494	14,746	3123	54 (43–66)	11,623	53 (41–66)	329
2017	497	14,803	3078	55 (43–67)	11,725	52 (40–66)	337
2018	463	14,901	3119	55 (44–68)	11,782	53 (40–66)	489
2019	450	15,047	3215	55 (44–68)	11,832	53 (41–67)	545
2020	421	13,161	2808	56 (44–67)	10,353	53 (41–67)	522
2021	394	13,267	2843	56 (44–68)	10,424	53 (41–67)	550
2022	354	13,069	2800	56 (44–68)	10,269	53 (41–67)	572
2023	343	13,396	2855	55 (43–67)	10,541	53 (41–66)	604

### Surgery for papillary thyroid carcinoma

**Fig. 6:**
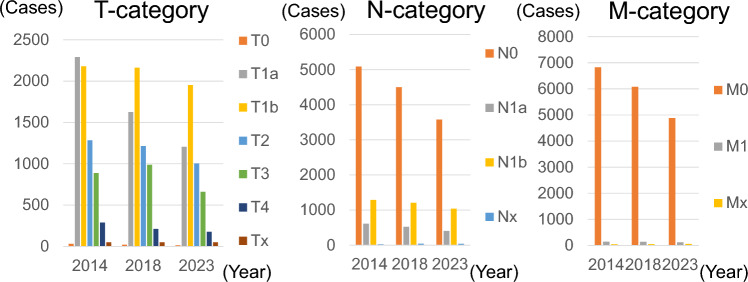
TNM classification of papillary thyroid cancer cases registered in 2014, 2018, and 2023. The numerical data used for plotting are given in Supplementary Table S1

**Fig. 7:**
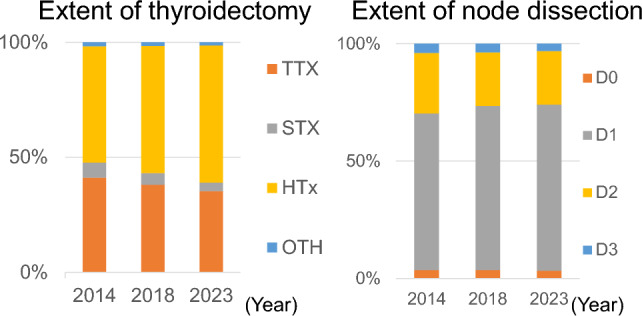
Extent of surgery for papillary thyroid cancer cases registered in 2014, 2018, and 2023 shown as the ratio to total surgery. The numerical data used for plotting are available in Supplementary Table S1. *TTX* total thyroidectomy, *STX* subtotal thyroidectomy, *HTx* hemithyroidectomy (lobectomy), *OTH* others

Figure 6 shows the preoperative TNM classification of cases of PTC surgery. There was a marked decrease in the number of surgeries for T1a lesions. There was also a marked decrease in the number of surgeries for N0 lesions, which was greater than the decrease in the number of surgeries for N1a and N1b lesions. There was a decrease in the number of surgeries for M0 lesions, but no change was observed in the number of surgeries for M1 lesions. In terms of surgical procedures, the number of total thyroidectomies decreased, and the proportion of hemithyroidectomies increased (Fig. 7). The number of subtotal thyroidectomies performed decreased significantly. The number of lymph node dissections also decreased, as did the number of D2 (lateral node dissection) and D3 (bilateral lateral node dissection), whereas the proportion of D1 (central node dissection) increased. No change was observed in the frequency of D0 (without node dissection). The final pathological diagnosis of the majority (> 95%) of lesions diagnosed preoperatively as PTC was papillary carcinoma, but other histological types were also observed (follicular thyroid carcinoma, 0.5%; poorly differentiated thyroid carcinoma, 0.6%; anaplastic thyroid carcinoma, 0.1%; and medullary thyroid carcinoma, 0.1%).

**Fig. 8:**
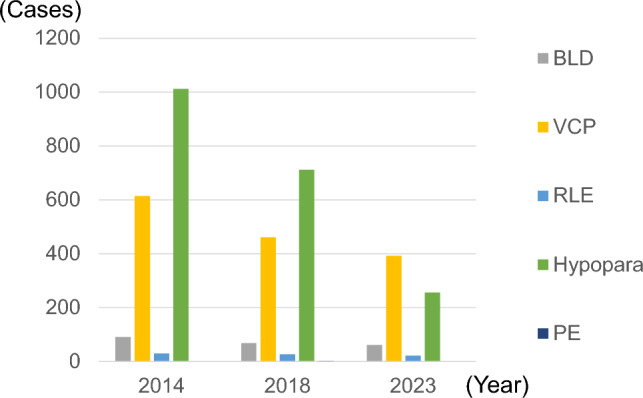
Number and frequency of the surgical complications reported after surgery for papillary thyroid cancer. The numerical data used for plotting are given in Supplementary Table S2

Figure 8 and supplementary Table S2 show the incidence of surgical complications of PTC surgery. Postoperative bleeding and laryngeal edema occurred in 1.2 and 0.4% of patients, respectively. Permanent vocal cord paralysis was recorded in 7.8% of patients. Permanent hypoparathyroidism was recorded in 10.6% of patients, but this decreased over time. Perioperative deaths were rare after surgery for PTC, with three cases recorded in 2014, two in 2018, and none in 2023. In total, there were five perioperative deaths (0.03%) among 18,690 surgical cases.

## Discussion

The number of thyroid cancers in Japan is increasing annually, with the majority being PTC [8]. Since the establishment of the Clinical Guidelines on the Management of Thyroid Tumors in 2010 [9], the JAES has consistently recommended non-surgical observation (active surveillance) for low-risk papillary microcarcinoma. While the number of surgeries has increased dramatically in some countries, such as South Korea and Cyprus [10], an increase has not been seen in Japan because of our consistent recommendations.

On the other hand, the number of facilities registered with the NCD and the number of registrations have been decreasing. The number of facilities registering cases of NCD is decreasing because of the nationwide decrease in endocrine surgeons and the consolidation of facilities performing thyroid surgery. This consolidation may be due, in part, to the beginning of the Board-Certified Endocrine Surgeon System of JAES in 2010, which has led to a decrease in surgeries outside certified training facilities. Moreover, in recent years, otolaryngologists and head and neck surgeons have taken over from endocrine surgeons as performers of thyroid surgery. Currently, the Japanese Society of Otolaryngology/Head and Neck Surgery is not a member of the NCD, so this study may indicate that data on thyroid surgery performed by otolaryngologists or head and neck surgeons are missing. Although we do not have accurate statistical data, there is a perception among medical professionals in clinical practice in Japan that the number of surgeons, including endocrine surgeons, is decreasing and that the burden of thyroid surgery is shifting to head and neck surgeons. We hope that this study will identify the shortage of endocrine surgeons and promote thyroid surgery performed by head and neck surgeons in the NCD registration project.

The present report demonstrates that the number of surgeries for malignant thyroid diseases has decreased significantly since the COVID-19 pandemic began in 2020. Thyroid cancer generally progresses slowly, and treatment is often not urgent. During the COVID-19 pandemic, general hospitals were forced to limit hospitalizations and surgeries. As a result, more urgent surgeries were prioritized, and thyroid surgeries decreased [11]. Thyroid cancer is often asymptomatic and discovered during screening; however, during the COVID-19 pandemic, people refrained from undergoing health checkups or visiting medical institutions. As a result, the number of thyroid cancers detected decreased, as did the number of surgeries. However, even in 2023, the number of surgeries for malignant diseases was trending downward. In addition to the above reasons for the recent increase in thyroid surgeries being performed by otolaryngologists and head and neck surgeons and the lack of NCD data on this, the recent international trend toward non-surgical surveillance of low-risk, minute PTCs may also account for the decline in the number of surgeries. The dramatic decline in the number of T1a PTC cases noted in this review demonstrates this. In Japan, active surveillance therapy without surgery for papillary thyroid microcarcinoma has been studied extensively since the 1990 s [12]. The latest 2024 Japanese guidelines recommend active surveillance for low-risk papillary cancer in adults, based on evidence, and the consensus among the committee members is high [8]. This may also explain the decrease in number of surgeries for T1a PTC. However, surgeries for benign diseases have returned to pre-COVID numbers, and it is interesting that only thyroid surgeries for malignant diseases are decreasing. Thyroid surgery for large benign goiters and benign hyperthyroidism is considered a highly specialized procedure and is presumably still being performed by endocrine surgeons rather than by otolaryngologists or head and neck surgeons.

In 2010, the first Japanese guidelines were published [9] and the Board-Certified Endocrine Surgeon System began in the same year, suggesting that the standardization of thyroid tumor management has progressed; however, this has not been verified until now. The guidelines were revised in 2018 [7] and 2024 [8]. The present data are the first to show the impact of the guidelines on practical thyroid surgical treatment in Japan.

Surgery for PTC has been consolidated into either total resection for high-risk lesions or hemithyroidectomy for low-risk lesions [7–9], and the choice of subtotal thyroidectomy, which was once mainstream and is an intermediate surgical procedure between total thyroidectomy and hemithyroidectomy, appears to have declined. The treatment of advanced thyroid cancer has changed over the past 15 years with the advent of drug therapy for radioactive iodine-refractory disease. It is possible that total thyroidectomy, instead of subtotal thyroidectomy, is becoming more popular as the initial treatment for advanced PTC, with the aim of allowing for subsequent treatment with radioactive iodine therapy and/or drugs. Furthermore, it is likely that the selection of high-risk lesions requiring total thyroidectomy is becoming stricter [9]. On the other hand, the proportion of lateral compartment lymph node dissections (D2, D3) has decreased. Since there has been no significant decrease in the number of cases of lateral compartment lymph node metastasis being found preoperatively (N1b), this tendency is thought to reflect the fact that the 2018 guidelines no longer recommend prophylactic lateral compartment lymph node dissection for low-risk papillary carcinoma [9].

Most thyroid cancers have a good prognosis and surgical invasiveness is relatively low grade; therefore, perioperative deaths are rare, except in cases of rapidly progressing anaplastic cancers. In this study, there were very few reported perioperative deaths after surgery for PTC. However, no changes were observed in the rate of perioperative complications observed after PTC surgery. The development and clinical application of intraoperative neuromonitoring and hemostatic devices has progressed. Moreover, as recommended in the 2024 guidelines [8], it is desirable to perform an objective evaluation of vocal cord movement using laryngeal fibers in all surgical cases. Regarding postoperative hypoparathyroidism, the registration diagnostic criteria changed in 2021; therefore, while no clear decrease was observed in 2023, no increase was observed either. In 2025, the definition of permanent hypoparathyroidism was changed to “iPTH below normal at 90 days.” Moreover, caution has been exercised about asphyxiation triggered by postoperative bleeding, and the latest 2024 guidelines stress the importance of ensuring perioperative safety [8]. This risk cannot be overlooked when performing thyroid surgery.

The current NCD case report form (CRF) is based on items that were considered necessary when the Japanese Society of Thyroid Surgery began collecting data more than 30 years ago and some of the items collected then may be outdated now. When analyzing the information collected for this report, it became apparent that some items or forms of data were technically difficult to analyze. The JAES NCD Committee plans to revise the CRF to make it more practical and useful.

In conclusion, we hope that this report will help improve the quality of thyroid surgery. The JAES is planning to develop and test procedures for new research questions in the field of thyroid surgery using NCD data. We anticipate that meaningful research topics will be published based on the present report.

## Supplementary Information

Below is the link to the electronic supplementary material.

Supplementary file1 (DOCX 18 KB)

Supplementary file2 (DOCX 17 KB)

## References

[1] Miyata H, Gotoh M, Hashimoto H, Motomura N, Murakami A, Tomotaki A, et al. Challenges and prospects of a clinical database linked to the board certification system. Surg Today. 2014;44:1991–9. 24849141 10.1007/s00595-013-0802-3

[2] Available from: http://www.ncd.or.jp/. Accessed 31 2022 Jul.

[3] Kajiwara Y, Takahashi A, Ueno H, Kakeji Y, Hasegawa H, Eguchi S, et al. National clinical database. Annual report on national clinical database 2020 for gastroenterological surgery in Japan. Ann Gastroenterol Surg. 2023;7:367–406. 10.1002/ags3.12662. 37152776 10.1002/ags3.12662 PMC10154850

[4] Matsumiya G, Sato Y, Takeuchi H, Abe T, Endo S, Hirata Y, et al. Thoracic and cardiovascular surgeries in Japan during 2020: annual report by the Japanese Association for Thoracic Surgery. Gen Thorac Cardiovasc Surg. 2024;72:61–94. 10.1007/s11748-023-01979-8. 38015364 10.1007/s11748-023-01979-8 PMC10766745

[5] Terui K, Tachimori H, Oita S, Fujiogi M, Fujishiro J, Hirahara N, et al. Influence of surgical volume on the mortality and morbidity of gastrointestinal perforation in children. Surg Today. 2024;54:419–27. 10.1007/s00595-023-02742-5. (**Epub 2023 Aug 24 PMID: 37615756**). 37615756 10.1007/s00595-023-02742-5

[6] Adachi Y, Asaga S, Kumamaru H, Kinugawa N, Sagara Y, Niikura N, et al. Analysis of prognosis in different subtypes of invasive lobular carcinoma using the Japanese National Cancer Database-Breast Cancer Registry. Breast Cancer Res Treat. 2023;201:397–408. 10.1007/s10549-023-07022-x. 37479943 10.1007/s10549-023-07022-x

[7] Ito Y, Onoda N, Okamoto T. The revised clinical practice guidelines on the management of thyroid tumors by the Japan Associations of Endocrine Surgeons: core questions and recommendations for treatments of thyroid cancer. Endocr J. 2020;6:669–717. 10.1507/endocrj.EJ20-0025. 10.1507/endocrj.EJ20-0025 32269182

[8] Sugitani I, Kiyota N, Ito Y, Onoda N, Hiromasa T, Horiuchi K, et al. The 2024 revised clinical guidelines on the management of thyroid tumors by the Japan Association of Endocrine Surgery. Endocr J. 2025. 10.1507/endocrj.EJ24-0644. 40058844 10.1507/endocrj.EJ24-0644 PMC12086281

[9] Takami H, Ito Y, Okamoto T, Onoda N, Noguchi H, Yoshida A. Revisiting the guidelines issued by the Japanese Society of Thyroid Surgeons and Japan Association of Endocrine Surgeons: a gradual move towards consensus between Japanese and western practice in the management of thyroid carcinoma. World J Surg. 2014;38:2002–10. 10.1007/s00268-014-2498-y. 24671301 10.1007/s00268-014-2498-y

[10] Li M, Maso LD, Pizzato M, Vaccarella S. Evolving epidemiological patterns of thyroid cancer and estimates of overdiagnosis in 2013–17 in 63 countries worldwide: a population-based study. Lancet Diabetes Endocrinol. 2024;12:824–36. 10.1016/S2213-8587(24)00223-7. 39389067 10.1016/S2213-8587(24)00223-7

[11] Ikeda N, Yamamoto H, Taketomi A, Hibi T, Ono M, Niikura N, et al. The impact of COVID-19 on surgical procedures in Japan: analysis of data from the National Clinical Database. Surg Today. 2022;52(1):22–35. 34783905 10.1007/s00595-021-02406-2 PMC8592826

[12] Miyauchi A, Ito Y, Fujishima M, Miya A, Onoda N, Kihara M, et al. Long-term outcomes of active surveillance and immediate surgery for adult patients with low-risk papillary thyroid microcarcinoma: 30-year experience. Thyroid. 2023;33(7):817–25. 10.1089/thy.2023.0076. 37166389 10.1089/thy.2023.0076 PMC10354707

