# Supplementary material for: Trends in thyroid surgery in Japan from 2014 to 2023: report on the National Clinical Database
Source: Surg Today. 2025 Nov 17;56(5):721–7. doi: 10.1007/s00595-025-03126-7 (PMC13090278; doi:10.1007/s00595-025-03126-7)
Supplement: Supplementary file 1 — Supplementary file1 (DOCX 18 KB) [file 595_2025_3126_MOESM1_ESM.docx]

**Supplementary Table S1.** TNM classification and extent of surgery for papillary thyroid carcinoma in 2014, 2018, and 2023

| Year | 2014 | 2018 | | 2023 |
| --- | --- | --- | --- | --- |
| T-category |  |  | |  |
| T0 | 32 | 21 | | 13 |
| T1a | 2291 | 1626 | | 1206 |
| T1b | 2180 | 2163 | | 1953 |
| T2 | 1283 | 1215 | | 1005 |
| T3 | 888 | 988 | | 661 |
| T4 | 289 | 211 | | 176 |
| Tx | 49 | 50 | | 50 |
| N-category |  |  | |  |
| N0 | 5089 | 4504 | | 3581 |
| N1a | 610 | 522 | | 405 |
| N1b | 1289 | 1206 | | 1040 |
| Nx | 24 | 42 | | 38 |
| M-category |  |  | |  |
| M0 | 6827 | 6082 | | 4885 |
| M1 | 143 | 142 | | 122 |
| Mx | 42 | 50 | | 57 |
| Extent of thyroidectomy | | |  | |
| Total thyroidectomy | 2941 | 2413 | | 1799 |
| Subtotal thyroidectomy | 466 | 321 | | 189 |
| Hemithyroidectomy | 3607 | 3506 | | 3041 |
| Others | 123 | 100 | | 68 |
| Extent of lymph node dissection | | |  | |
| D0 | 246 | 217 | | 160 |
| D1 | 4658 | 4298 | | 3482 |
| D2 | 1797 | 1399 | | 1117 |
| D3 | 277 | 229 | | 156 |
