# Supplementary material for: Trends in thyroid surgery in Japan from 2014 to 2023: report on the National Clinical Database
Source: Surg Today. 2025 Nov 17;56(5):721–7. doi: 10.1007/s00595-025-03126-7 (PMC13090278; doi:10.1007/s00595-025-03126-7)
Supplement: Supplementary file 2 — Supplementary file2 (DOCX 17 KB) [file 595_2025_3126_MOESM2_ESM.docx]

**Supplementary** **Table S2.** Number and frequency of surgical complications

|  | 2014 | 2018 | 2023 | total |
| --- | --- | --- | --- | --- |
| Bleeding | 91 (1.3%) | 68 (1.1%) | 61 (1.2%) | 220 (1.2%) |
| Vocal cord paralysis | 614 (8.6%) | 461 (7.2%) | 392 (7.6%) | 1467 (7.8%) |
| Laryngeal edema | 30 (0.4%) | 27 (0.4%) | 22 (0.4%) | 79 (0.4%) |
| Hypoparathyroidism | 1012(14.1%) | 712 (11.2%) | 256 (5.0%) | 1980 (10.6%) |
| Pulmonary embolism | 0 (-) | 3 (0.0%) | 0 (-) | 3 (0.0%) |
| Surgical mortality ^1)^ | 3 (0.0%) | 2 (0.0%) | 0 (-) | 5 ((0.0%) |
| Total number | 7171 | 6379 | 5140 | 18690 (100%) |

Surgical mortality included both 30-day and in-hospital mortality, and the NCD system ensured follow-up during hospitalization for at least 90 days after surgery
